# Crabbed Age and Youth

**Published:** 1901-12-21

**Authors:** 


### "Crabbed Age and Youth."

We have lately called attention to the increasing frequency of longevity in this country, and to the excellent advice given by Sir Henry Thompson with regard to the way in which old age may best be nourished. In this advice, however, the general question of how the old should live is not exhausted, and it is one which, in view of the continually larger and larger share taken by the aged in the transaction of public business, and even in the government of the country, cannot be said to be unimportant. Within the compass of quite modern history the attainment of a period of life at which our contemporaries are still active was regarded as affording a sufficient reason for repose. There lies before us a somewhat typical letter, written from a fashionable watering place a hundred years ago by an octogenarian clergyman, whose years alone had seemed to him to furnish an adequate reason for resigning his living and for awaiting his end in absolute tranquility. "This place," he writes, "is very full, and here are innumerable characters as well as faces, affording abundant opportunity for amusement, satire, and ridicule. I hear and see much, but say little; am struck with observing the indiscretion of the young, and the juvenile affectation of the old." There is here a soit of aloofness from surrounding interests, an indifference to prevailing topics of thought or of conversation, an assumption of the position of the merely contemplative observer, which we should hardly find paralleled in our own day. And yet the writer, like the "old man" in Jean Ingelow's charming poem, was one "whose story did not shame him," and whose life had been full and vigorous enough in what he conceived to be its day. The way in which we now differ from him is chiefly in respect of his ungrudging acceptance of the view that the evening of that day had come. The octogenarians now with us are largely continuing in the pursuits to which their lives have been devoted, or have exchanged these pursuits for others perhaps equally engrossing. They are expected to confer upon mankind the results of their long experience, or to temper the counsels of youth or middle age by the maturity of their wisdom.

And so, if old age in this our day is called upon still to keep its lamp burning, and its loins girded, it must surely evoke all possible aid from physiology for the attainment of a full knowledge of the differences which distinguish it from youth, and of the ways in which its life should be so ordered as to conduce to the attainment of the ends desired. Sir Henry Thompson has instructed us concerning feeding; and the recently published memoir of Sir James Paget tells us something with regard to sleep. Possibly, said Sir James, referring to one of the proverbs more familiar to the last generation than to the present, six hours' sleep may be enough for a fool, but a man wants eight. But the sleep necessary for the aged can seldom be taken, as may that of the young, during only one period of the twenty-four hours; and, like their meals, should be more divided than was at one time either necessary or desirable. An old man can but seldom sleep all night. "He shall rise up" says the Preacher, "at the voice of the bird;" and it is clear that he may prudently devote what Longfellow described as "the children's hour" to obtaining the refreshment of another instalment of Nature's sweet restorer. Sir James Paget is very interesting and instructive in his description of the way in which the old man should obtain self-knowledge of his growing incapacities by keeping watch over the altered character of some of his acts, over the half mile less which he can accomplish in an hour, over the gradual curtailment of the range in both directions of his voice, and over the way in which he should seek to adapt his efforts to his failing powers. Such self-observation might perhaps be trusted to afford immunity from some of what may perhaps be called the vices of old age; the one story repeated an uncountable number of times, the intellect so imbedded in a groove that it cannot be extricated by the advent of new knowledge.

Sir Andrew Clark was accustomed to define old age as the period of life at which a man no longer adjusted himself to his environment; and to illustrate the definition by saying that he regarded as old a doctor who expressed himself contumeliously with regard to microbes, or who, like one of his own contemporaries, spoke of the tubercle bacilli as "lung-bugs." Perhaps, as regards action, the sum of practical wisdom in the matter may be reached by Sir James Paget's admonition that the old man must not only say of his intentions "I will," but "I will *now*," in recognition that the limit of his activities in time is approaching, and that none can say how much of any work that he defers it may ever be within his power to accomplish.

